# AI-Generated Draft Replies Integrated Into Health Records and Physicians’ Electronic Communication

**DOI:** 10.1001/jamanetworkopen.2024.6565

**Published:** 2024-04-15

**Authors:** Ming Tai-Seale, Sally L. Baxter, Florin Vaida, Amanda Walker, Amy M. Sitapati, Chad Osborne, Joseph Diaz, Nimit Desai, Sophie Webb, Gregory Polston, Teresa Helsten, Erin Gross, Jessica Thackaberry, Ammar Mandvi, Dustin Lillie, Steve Li, Geneen Gin, Suraj Achar, Heather Hofflich, Christopher Sharp, Marlene Millen, Christopher A. Longhurst

**Affiliations:** 1 Department of Family Medicine, University of California San Diego School of Medicine, La Jolla; 2 Department of Medicine, University of California San Diego School of Medicine, La Jolla; 3 Division of Ophthalmology Informatics and Data Science, Viterbi Family Department of Ophthalmology and Shiley Eye Institute, University of California San Diego School of Medicine, La Jolla; 4 Division of Biostatistics, University of California San Diego Herbert Wertheim School of Public Health and Human Longevity Science, La Jolla; 5 University of California San Diego School of Medicine, La Jolla; 6 Department of Anesthesiology, University of California San Diego School of Medicine, La Jolla; 7 Department of Obstetrics and Gynecology, University of California San Diego School of Medicine, La Jolla; 8 Department of Psychiatry, University of California San Diego School of Medicine, La Jolla; 9 Department of Medicine, Stanford School of Medicine, Stanford, California; 10 Department of Pediatrics, University of California San Diego School of Medicine, La Jolla

## Abstract

**Importance:**

Timely tests are warranted to assess the association between generative artificial intelligence (GenAI) use and physicians’ work efforts.

**Objective:**

To investigate the association between GenAI-drafted replies for patient messages and physician time spent on answering messages and the length of replies.

**Design, Setting, and Participants:**

Randomized waiting list quality improvement (QI) study from June to August 2023 in an academic health system. Primary care physicians were randomized to an immediate activation group and a delayed activation group. Data were analyzed from August to November 2023.

**Exposure:**

Access to GenAI-drafted replies for patient messages.

**Main Outcomes and Measures:**

Time spent (1) reading messages, (2) replying to messages, (3) length of replies, and (4) physician likelihood to recommend GenAI drafts. The a priori hypothesis was that GenAI drafts would be associated with less physician time spent reading and replying to messages. A mixed-effects model was used.

**Results:**

Fifty-two physicians participated in this QI study, with 25 randomized to the immediate activation group and 27 randomized to the delayed activation group. A contemporary control group included 70 physicians. There were 18 female participants (72.0%) in the immediate group and 17 female participants (63.0%) in the delayed group; the median age range was 35-44 years in the immediate group and 45-54 years in the delayed group. The median (IQR) time spent reading messages in the immediate group was 26 (11-69) seconds at baseline, 31 (15-70) seconds 3 weeks after entry to the intervention, and 31 (14-70) seconds 6 weeks after entry. The delayed group’s median (IQR) read time was 25 (10-67) seconds at baseline, 29 (11-77) seconds during the 3-week waiting period, and 32 (15-72) seconds 3 weeks after entry to the intervention. The contemporary control group’s median (IQR) read times were 21 (9-54), 22 (9-63), and 23 (9-60) seconds in corresponding periods. The estimated association of GenAI was a 21.8% increase in read time (95% CI, 5.2% to 41.0%; *P* = .008), a −5.9% change in reply time (95% CI, −16.6% to 6.2%; *P* = .33), and a 17.9% increase in reply length (95% CI, 10.1% to 26.2%; *P* < .001). Participants recognized GenAI’s value and suggested areas for improvement.

**Conclusions and Relevance:**

In this QI study, GenAI-drafted replies were associated with significantly increased read time, no change in reply time, significantly increased reply length, and some perceived benefits. Rigorous empirical tests are necessary to further examine GenAI’s performance. Future studies should examine patient experience and compare multiple GenAIs, including those with medical training.

## Key Points

**Question:**

Would access to generative artificial intelligence–drafted replies correlate with decreased physician time on reading and replying to patient messages, alongside an increase in reply length?

**Findings:**

In this quality improvement study including 122 physicians, generative AI-drafted replies correlated with increased message read time, no change in reply time, and significantly longer replies. Physicians valued AI-generated drafts as a compassionate starting point for their replies and also noted areas for improvement.

**Meaning:**

These findings suggest generative AI was not associated with reduced time on writing a reply but was associated with longer read time, longer replies, and perceived value in making a more compassionate reply.

## Teaser

This quality improvement study investigates the association between generative artificial intelligence–drafted replies for patient messages and physician time spent on answering messages and the length of replies.

## Introduction

Electronic messaging in electronic health records (EHRs) is a major source of physician burnout. Prior studies found significant time spent answering messages^1,2^ and associated stress.^3^ Strategies to address this challenge include triaging messages by care teams,^4^ charging fees,^4,5^ and using templated responses.^6^ Published work suggested that generative artificial intelligence (GenAI) could potentially extend this toolset by drafting replies.^7^ UC San Diego Health is among the first health systems to collaborate with an EHR vendor (Epic Systems) to implement GenAI natively in the EHR.^8^ Timely tests of AI’s applications in medicine are urgently needed.^9^ We evaluated how implementing GenAI may be associated with patient-physician communication.

## Methods

This quality improvement (QI) study was deemed nonhuman participants research and exempt from institutional review board review by the University of California San Diego Aligning and Coordinating Quality Improvement, Research and Evaluation Committee. Answering the baseline survey served as implied consent to participate in the project. This study is reported following the Standards for Quality Improvement Reporting Excellence (SQUIRE) reporting guideline.^10^

### Integration of GenAI Draft Replies in the Clinical Workflow

Four types of patient medical advice requests were eligible for GenAI drafts: refills, results, paperwork, and general questions. These types of patient messages were reviewed by GenAI, which generated a draft reply to present to the physician’s inbasket. The time between when a patient message was received and a GenAI draft appeared was typically less than 1 minute. This time was also capped at 4 minutes. Therefore, if a draft was not provided in 4 minutes, the patient message would be shown to physicians without a GenAI draft reply. In 1 day during the week of January 22, 2024, GenAI drafts were provided after a mean of 55 seconds from receiving patient messages (median [range] 57 [25-220] seconds) (email from Matthew Wiese, BA, Epic, February 2, 2024).

### Recruitment and Enrollment of QI Study Participants

After activating the GenAI feature for Family Medicine and General Internal Medicine, attending physicians in those departments were invited to participate via email and presentations in department meetings between June 16 and July 12, 2023. Up to 2 follow-up email reminders were sent to encourage participation. A participant identification number was assigned to each participant and used to perform randomization. Fifty-two physicians (43% participation rate) volunteered.

A tip sheet was distributed to guide participants. eFigure 1 in Supplement 1 displays the tip sheet. Item 5 in the tip sheet included a screen shot of the patient message box and the generated draft box in the EHR. It contained clear language on the choice to “Start with Draft” or “Start Blank Reply.” The “Start with Draft” button also created a UC San Diego Health system message quick action (a shortcut) that included an automated sign off with the name of the sender. eFigure 2 in Supplement 1 displays screenshots of a GenAI draft reply which was used by a physician and the reply she sent. “Take care, [name of doctor], MD” was the automated sign off. To ensure transparency, the following statement was provided by default: “Part of the message was generated automatically and was reviewed and edited by [name of doctor].”

### Design of QI Study

A modified waiting list randomized QI study was chosen. Observation of physicians’ management of electronic messages took place over 3 periods: time 0 (T0, or baseline), which encompassed the 3 weeks before entry to the pilot, time 1 (T1), which referred to 3 weeks after entry, and time 2 (T2), which covered 4 to 6 weeks after entry.

Participants were randomized into 2 groups: immediate (25 participants) and delayed (27 participants) activation. The delayed group was activated 21 days after the immediate group. The intervention affected the immediate group for both T1 and T2, and the delayed group for T2 only. Physicians in these departments who did not participate in the pilot (70 physicians) served as contemporary controls. No GenAI activation occurred in the control group. Participants were surveyed at baseline, 3 weeks, and 6 weeks after entering the pilot.

This design was motivated by 3 reasons. First, by comparing outcomes between the group that received the intervention immediately and the group that received it after a delay, factors such as participant expectations and natural fluctuations in the outcomes could be controlled. Second, in projects where a potentially beneficial intervention was being investigated, it might be unethical to deny participants the intervention. A waiting list design allowed all volunteers to eventually receive the intervention while still providing a control group. It could also help reduce dropout. Knowing they would receive the intervention might encourage physicians to remain engaged. Third, the additional contemporary control group could enhance the validity and interpretability of the findings. As a contemporary control group, nonparticipants in departments where the GenAI feature was available allowed us to compare the outcomes not only with those on the waiting list but also with a group that was not receiving any intervention during the waiting period. This helped ensure that any observed changes in the intervention group were not simply due to the passage of time or other external factors. Furthermore, waiting list control groups can sometimes introduce bias, as participants are aware that they will eventually receive the intervention, which could affect their behavior during the waiting period. Having a contemporary control group that did not receive any intervention could help mitigate this bias by providing a comparison group that was not influenced by the anticipation of receiving intervention.

### Sample Size and Power

Power calculation was based on the goal of achieving a 20% reduction in time spent on answering electronic messages with 80% power. A 2-sample *t* test power calculation with α = .05 suggested that 46 participants (23 per group) were needed.

### Outcomes Assessment

This report focuses on (1) time spent reading messages, (2) time spent replying to messages, (3) length of replies, and (4) physician likelihood to recommend GenAI drafts on a scale of 0 to 10. Time spent answering patient messages came from the user access log, which tracks discrete, time-stamped actions within the section of the EHR that was accessed.^11,12^ The user access log data allow measurement of the time spent on a task by calculating the difference between the time when the task ended and the time when it began.^11^ Read time was calculated as the difference between when a reply to the patient was started by a user clicking either “reply,” “start with draft,” or “start blank reply,” and when that user most recently selected the message. Reply time was the difference in time when a reply was started (defined as previously described) and when the reply was sent. Reply length was measured by the number of characters in each reply. Included in the survey (eMethods in Supplement 1) was a likelihood to recommend question, similar to a net promoter score question commonly used for assessing experience.^13^ Participant race and ethnicity options were defined by the QI team and self-selected by participants and were measured to enable comparison of the immediate and delayed groups.

### Statistical Analysis

The responses were summarized as median and IQR by group and period. Under intention-to-treat, the statistical analysis used a linear mixed-effects model for each of the 3 outcomes: log-transformed read time, log reply time, and log reply length, as a function of the study group, study period, and their interaction, and included a physician-specific random intercept. The group differences were estimated on the logarithmic scale, and then back-transformed and reported as proportional differences between groups. Wald 95% CIs were also reported. Hypothesis tests for group comparisons used the Wald test with significance level set at α = .05.

When assessing the intervention effect across all 3 groups combined, a model term for intervention was included for the immediate group at T1 and T2 and for the delayed group for T2. This term reflects the time when the intervention was in effect, as opposed to the control condition. It was included as a covariate in a linear mixed-effects model. This model also included a main effect for group to account for baseline (T0) differences between groups and a main effect for period to account for time trends over time affecting all groups equally. Analyses were performed in R version 4.3.2 (R Project for Statistical Computing). Data were analyzed from August to November 2023.

#### Qualitative Analysis

Thematic analysis of survey responses identified themes related to the underlying reason for the stated likelihood to recommend GenAI draft replies to colleagues (see survey questions provided in eMethods in Supplement 1). Researchers and publicly available ChatGPT 3.5 analyzed the narrative reasons. Consensus on themes was reached following iterative analysis.

## Results

Fifty-two physicians participated in this QI study, with 25 randomized to the immediate activation group and 27 randomized to the delayed activation group. Demographics are provided in the eTable in Supplement 1. There were 18 female participants (72.0%) in the immediate group and 17 female participants (63.0%) in the delayed group; the median age range was 35-44 years in the immediate group and 45-54 years in the delayed group; the median duration of employment was 6-10 years in the immediate group, and 11-15 in the delayed group. In the immediate group, 16 of 25 physicians (64.0%) were White, 7 of 25 physicians (28.0%) were Asian, and 2 of 25 physicians (8.0%) were other races and ethnicities; in the delayed group, 14 of 27 physicians (51.9%) were White, 7 of 27 physicians (25.9%) were Asian, 1 of 27 physicians (3.7%) were Hispanic, and 5 of 27 (18.5%) were other races and ethnicities (the other race and ethnicity group included 4 participants who were 2 or more races, and 3 participants who preferred not to answer). There were no significant differences in the groups’ demographic characteristics. Demographic information is not available on physicians in the contemporary control group.

### Time Spent on Reading, Replying to Patient Messages, and Reply Length

**Figure 1.**
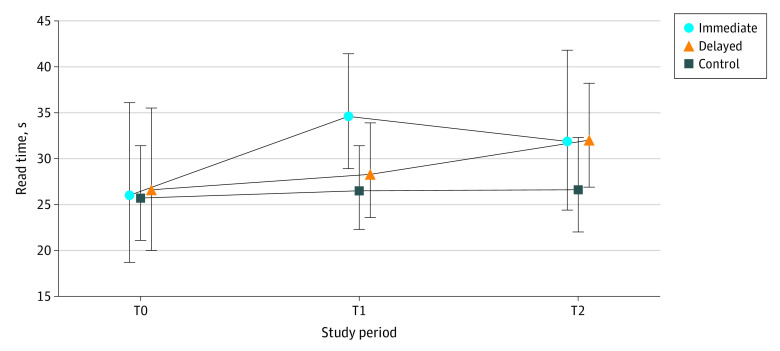
Read Time per Message (in Seconds) for Each Group and Study Period Symbols indicate geometric means and whiskers indicated 95% CIs. T0 indicates preactivation period, 1 to 21 days before randomization day; T1, 0 to 20 days post randomization and activation (immediate activation group), or 0 to 20 days after randomization date (delayed group); T2, 21 to 41 days post activation (immediate group), or 0 to 20 days post activation (delayed group).

**Figure 2.**
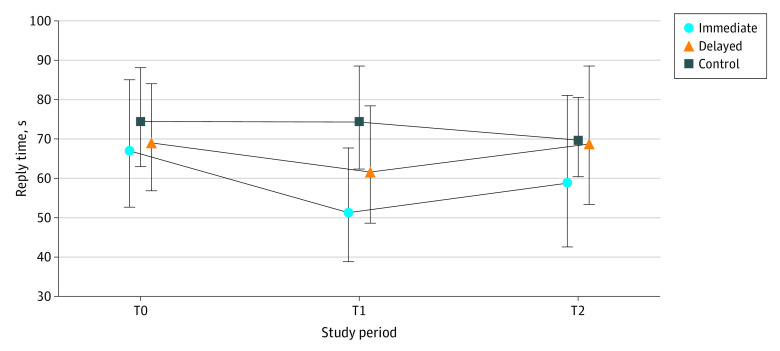
Reply Time per Message (in Seconds) for Each Group and Study Period Symbols indicate geometric means and whiskers indicated 95% CIs. T0 indicates preactivation period, 1 to 21 days before randomization day; T1, 0 to 20 days post randomization and activation (immediate activation group), or 0 to 20 days after randomization date (delayed group); T2, 21 to 41 days post activation (immediate group), or 0 to 20 days post activation (delayed group).

**Figure 3.**
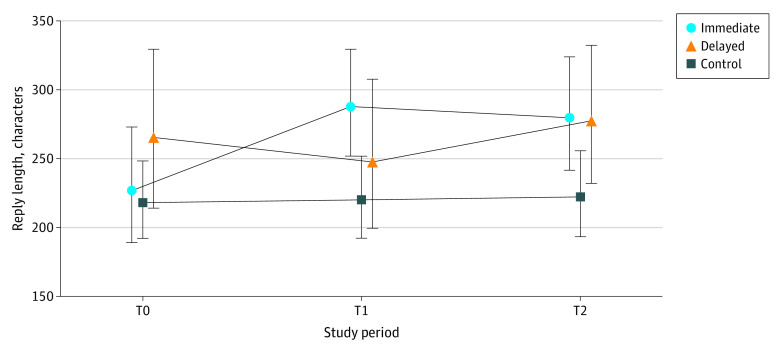
Reply Length for Each Group and Study Period Symbols indicate geometric means and whiskers indicated 95% CIs. T0 indicates preactivation period, 1 to 21 days before randomization day; T1, 0 to 20 days post randomization and activation (immediate activation group), or 0 to 20 days after randomization date (delayed group); T2, 21 to 41 days post activation (immediate group), or 0 to 20 days post activation (delayed group).

Among the 10 679 replies to patient messages examined, the median (IQR) read times among those in the immediate group were 26 (11-69), 31 (15-70), and 31 (14-70) seconds at T0, T1 and T2, respectively. The delayed group’s median (IQR) times were 25 (10-67), 29 (11-77), and 32 (15-72) seconds in T0, T1, and T2, respectively. The control group spent 21 (9-54), 22 (9-63), and 23 (9-60) seconds in corresponding periods. Descriptive statistics on read time, reply time, and reply length are shown in Table 1. Figure 1, Figure 2, and Figure 3 illustrate geometric means and 95% CIs of each outcome.

**Table 1.** Time Spent Answering Patient Messages (in Seconds) and Reply Length Across Physician Groups Over 3 Periods

Period	Immediate activation (n = 25 physicians)		Delayed activation (n = 27 physicians)		No activation (n = 70 physicians)	
	No.	Median (IQR)	No.	Median (IQR)	No.	Median (IQR)
Read time, s						
Time 0^a^	713	26 (11-69)	813	25 (10-67)	2154	21 (9-54)
Time 1^b^	788	31 (15-70)	781	29 (11-77)	1908	22 (9-63)
Time 2^c^	768	31 (14-70)	746	32 (15-72)	1988	23 (9-60)
Reply time, s						
Time 0^a^	713	70 (38-122)	813	71 (37-128)	2154	58 (28-124)
Time 1^b^	788	60 (32-126)	781	64 (30-123)	1908	56 (28-114)
Time 2^c^	768	65 (28-119)	746	71 (32-127)	1988	60 (28-127)
Reply length, characters						
Time 0^a^	713	214 (124-361)	813	276 (154-457)	2154	210 (115-371)
Time 1^b^	788	304 (175-528)	781	252 (146-451)	1908	211 (117-379)
Time 2^c^	768	276 (150-463)	746	287 (157-488)	1988	212 (113-382)

^a^ Preactivation period, 1 to 21 days before randomization day.

^b^ Zero to 20 days post randomization and activation (immediate activation group), or 0 to 20 days after randomization date (delayed group).

^c^ Twenty-one to 41 days post activation (immediate group), or 0 to 20 days post activation (delayed group).

The estimated change between GenAI draft and read time was a statistically significant 21.8% increase (95% CI, 5.2% to 41.0%; *P* = .008). The change in reply time was −5.9% (95% CI, −16.6% to 6.2%; *P* = .33), which was not statistically significant. The change in reply length showed a statistically significant increase of 17.9% (95% CI, 10.1% to 26.2%; *P* < .001).

### Examples of GenAI-Drafted Replies and Replies Sent by Physicians

To provide context for quantitative results, 3 examples of GenAI-drafted replies and actual replies by physicians are provided. The first example is shown in eFigure 2 in Supplement 1. The examples suggest that while the GenAI draft’s first paragraph was used by the physician without change, the physician personalized the reply by adding more patient-specific information in the second paragraph. In the second example, the GenAI-drafted reply was 103 words and 498 characters without spaces, and can be seen here:

*Hello [patient name], I’m glad to hear that your knee pain has improved on its own. Regarding your elbow pain, it’s important that we discuss your symptoms and evaluate the best course of action. It may be necessary to consider an x-ray or a referral for physical therapy, but we can determine that during your appointment on [date]. As for your test results, no results are available at this time. When results become available, you can view them in MyChart under the “Test Results.” If you have any further concerns or questions, please don’t hesitate to schedule an additional appointment to discuss them in person. Sincerely, [name of Epic employee] Part of this message was generated automatically and was reviewed and edited by [name of Epic employee].*

The physician’s reply was 59 words and 252 characters without spaces, and is as follows:

Hello [patient name], I’m glad to hear that your knee pain has improved on its own. Regarding your elbow pain, unless you had an acute injury like a fall or an impact, it is unlikely that the x-rays will show very much. I would much prefer you make or keep the [date] appointment for a good examination before exposing you to x-rays. Best regards, [physician name], MD [Signature Derived From Controlled Access Password] Part of this message was generated automatically and was reviewed and edited by [physician name, MD].

Similarities between the 2 replies included acknowledging the improvement in the patient’s knee pain, addressing the patient’s concerns about elbow pain and providing guidance on the next steps, and expressing concern for the patient’s well-being. However, substantive differences also existed. The GenAI draft was providing clinical advice (“consider an X-ray or physical therapy”), which was outside of its scope. The physician wrote a more succinct and direct reply with a more cautious approach, prioritizing a thorough physical examination first. Only the first sentence from the draft was retained by the physician.

In the third example, the GenAI-drafted reply was 160 words and 767 characters without spaces and can be seen here:

*Hello [patient name], I hope you had a great Fourth of July weekend as well! I’m glad to hear that the lab billing issue has been resolved. Regarding your … prescription, it’s good to know that you’ve been tolerating it well and have seen significant weight loss. Since you mentioned that the pharmacy has the 1.75 mg dose available, I can write a new prescription for that dosage. However, I would recommend scheduling an appointment to discuss this change further and ensure it’s the best course of action for you. It’s great that you’ve received your recent COVID vaccine, and I’ll make sure to add that information to your chart. We can discuss the new phenomena vaccine and the shingles vaccine during your next appointment. As for any further labs, I will review your records and let you know if any additional tests are needed before your appointment on [date]. Safe travels to Thailand, and I look forward to seeing you when you return!*

The physician’s reply was 173 words and 804 characters without spaces, and is as follows:

Hello [patient name], I hope you had a great Fourth of July weekend as well! I’m glad to hear that the lab billing issue has been resolved. Regarding your … prescription, it’s good to know that you’ve been tolerating it well and have seen significant weight loss. Since you mentioned that the pharmacy has the 1.75 mg dose available, I was just worried about the strength of it as it cannot be split (according to pharmacy, I have not seen personally). So, what are your thoughts on the dose, which is fairly high and side effect risk? It’s great that you’ve received your recent COVID vaccine, when you come in, we can add that information to your chart. We can discuss the new vaccine and the shingles vaccine during your next appointment. As for any further labs, I did review your records and think none are needed before your appointment on [date], but is also up to you, officially due in Sept/October timeframe. Safe travels to Thailand, and I look forward to seeing you when you return! Take care, [physician name], MD *Part of this message was generated automatically and was reviewed and edited by [physician name], MD.*

While both replies demonstrated a caring attitude to the patient’s concerns, the physician’s reply contained greater details regarding medication concerns and offered a more collaborative approach to decision-making regarding further laboratory testing. The physician retained the pleasantries in the GenAI draft and made many substantive changes.

### Likelihood to Recommend

A survey asked participants regarding their likelihood of recommending GenAI drafted replies (on a scale of 0 to 10) to colleagues and the reason for their recommendation score. Three weeks after activation of the immediate group, 15 of 25 participants completed the survey. Six weeks after activation of the immediate group, 19 participants responded to the survey. Three weeks after the delayed group was activated, 21 of 27 participants responded. Summarized in Table 2, the comments related to perceived values of the GenAI-drafted replies ranged from finding potential benefits in starting drafts and infusing empathy into replies, despite the need for editing, to ineffective, or being overly focused on recommending appointments. While some were put off by the “overly nice” tone of AI-drafted replies, one physician stated: “I do really like the ‘empathic tone’ of the messages—it makes me feel better sending it....reminds me of Lincoln: ‘by the better angels of our [inbox] nature.’”

**Table 2.** Themes and Quotes From Physician Likelihood to Recommend Generative Artificial Intelligence (AI) Drafted Replies to Their Colleagues, on a Scale of 0 to 10 Where 0 is Not At All and 10 is Definitely

Likelihood to recommend	Quote
Likelihood: 9 or 10	
Tone and value: acknowledgment of the robotic tone of AI replies, recognizing their role in initiating patient interactions, and serving as a valuable baseline.	“Though the replies sound very robotic still, they’re extremely helpful for generating the baseline response to what you’d want to say to a patient.”
Potential for improvement and mimicry of physician language: anticipation for AI-generated replies to improve and emulate the communication style of individual physicians, enhancing personalization and human-like interactions.	“I can’t wait for them to get even better, to the point where they can mimic each physician’s language/tone.”
AI replies’ place and role: recognition of AI-generated replies’ valuable role in health care workflows, aiding in workload management and effective patient communication, and contributing to workflow efficiency.	“I think AI responses have its place. [I] worry about inaccuracies that I may miss due to busy workload. I have been very impressed with [a] few of the responses.”
Hope for reduced supervision: expressing hope for AI advancements leading to reduced supervision, envisioning a future where AI can function autonomously while maintaining high-quality patient communication.	“Great initiative which requires supervision. Hopefully there would be time when minimal supervision would be needed.”
Likelihood: 7 or 8	
Tone and empathy: recognition of AI-generated replies for their kind and empathetic tone, aiding in maintaining respectful and caring interactions with patients.	“Helpful in drafting responses, provides more empathy into a response without me taking time to type it all out.”
Time savings and future expectations: appreciation for saving time and enhancing efficiency by initiating tailored drafts swiftly, compared with starting from scratch; optimism about future enhancements.	“Not perfect but decreases time I spend on it and has a kind tone.”
	“While not perfect, I think there have been a good number of cases where I use the draft as a starting point. I expect the AI responses to get better over time.”
Alleviating pressure to address patient concerns online only: perceived relief from the pressure of responding solely through MyChart, with AI aiding appropriate recommendations for in-person evaluations when necessary, thus lessening workload.	“AI generated messages often appropriately recommend that the patient be evaluated in person for specific concerns. Sometimes clinicians feel pressured to deal with patient concerns by MyChart alone. The use of AI generated messages can take away this pressure.”
Recommendation to colleagues: general endorsement of AI-generated draft replies to colleagues, emphasizing its potential benefits in starting drafts and infusing empathy into responses, despite the need for some editing.	“I generally would recommend auto-generated draft replies to colleagues because it seems to be net even—it may be helpful to start a draft, but most of the time, I am editing the replies, so it is not completely/automatically helpful.”
Likelihood: 0-6	
Tone and language: critique of AI-generated replies for being excessively polite, formal, impersonal, and not aligning with the desired direct and concise tone in patient interactions.	“Messages were too nice and wordy, but sometimes offered good advice.”
	“Not personalized to the specific patient. I tend to personalize my response in different ways depending on the patient.”
Efficiency and time savings: recognition of AI replies as helpful starting points, but often requiring extensive editing, reducing potential time savings compared with drafting from scratch.	“I found I used them most when I was covering for other providers and did not know the patients as well so did not need to provide as customized of a message.”
Challenges in clinic population and workload: noting challenges in AI replies’ applicability, particularly regarding urgent and specific patient needs, where recommending appointments may not be appropriate or feasible.	“It ended up creating more work for me and ultimately always recommended scheduling an appointment. Our clinic is largely populated with persons who are struggling with multimorbidity and SDOH [social determinants of health] and need urgent assistance rather than a future appointment.”
Improvement and future potential: acknowledgment of AI potential, urging significant improvement in understanding patient queries, offering accurate information, and considering context for appropriate responses.	“I think it has potential, but is not anywhere near where it needs to be to be useful.”

## Discussion

This initial GenAI pilot suggests having access to GenAI-generated draft replies was associated with a significant increase in read time, no change in reply time, and significantly longer replies. The uptick in read time may be attributable to the need to read both the patient’s original message and the draft reply. It might suggest effort in scrutinizing drafts for hallucinations,^14^ which can occur due to the complexity of the GenAI model and its training data, leading to unexpected or nonsensical outputs.

While some physicians clearly perceived GenAI’s value, including reduced cognitive burden due to having a draft infused with empathy to start their reply, opportunities for enhancement lie in achieving greater personalization to align with physicians’ tone and better decisions on whether to recommend a visit. Additional examination of empathic opportunities^15^ and empathic replies^7^ is warranted in view of the noteworthy increase in reply length. It is plausible that the additional characters were those extra personal touches that are highly valued by patients.^16^

GenAI’s current performance suggests that human input is still essential.^14^ Additional efforts are also needed to compare multiple GenAIs, including those with specific medical training, to improve relevance of draft replies and identify validated GenAIs to scale high-quality service to the public.^17^

### Limitations

This study has limitations. Only 1 academic health system’s early experience in using GenAI in primary care is reported here. Findings may not generalize to GenAI-EHR customizations in other organizations, to other medical specialties, or over a longer time horizon.

## Conclusions

In this quality improvement study of generative AI in medicine, we found that having access to GenAI-generated draft replies was associated with a significant increase in read time, no change in reply time, and significantly longer replies. A balanced approach that focuses on GenAI’s present capabilities and limitations is necessary to realize its potential in medicine while avoiding unrealistic expectations. Future studies should focus on patient experiences of these AI-generated messages. It would be important to assess if patients notice and appreciate the increased empathy and length, even if they are told that parts of the message were generated by AI.

## Notes

Supplement 1. **eFigure 1. **Tip Sheet Used for Training **eFigure 2. **Screen Shots of a Generated Draft Reply and the Physician’s Reply **eMethods. **Survey Questions in Postentry Survey **eTable. **Demographic Characteristics of Participants

Supplement 2.
Data Sharing Statement
