# Supplementary material for: AI-Generated Draft Replies Integrated Into Health Records and Physicians’ Electronic Communication
Source: JAMA Netw Open. 2024 Apr 15;7(4):e246565. doi: 10.1001/jamanetworkopen.2024.6565 (PMC11019394; doi:10.1001/jamanetworkopen.2024.6565)
Supplement: Supplement 1. — eFigure 1. Tip Sheet Used for Training eFigure 2. Screen Shots of a Generated Draft Reply and the Physician’s Reply eMethods. Survey Questions in Postentry Survey eTable. Demographic Characteristics of Participants [file jamanetwopen-e246565-s001.pdf]

Supplemental Online Content

Tai-Seale M, Baxter SL, Vaida F, et al. AI-generated draft replies integrated into health records and physicians' electronic communication. *JAMA Netw Open*. 2024;7(4):e246565. doi:10.1001/jamanetworkopen.2024.6565

eFigure1. Tip Sheet Used for Training
eFigure2. Screen Shots of a Generated Draft Reply and the Physician's Reply
eMethods. Survey Questions in Postentry Survey
eTable. Demographic Characteristics of Participants

This supplemental material has been provided by the authors to give readers additional information about their work.

© 2024 Tai-Seale M et al. *JAMA Network Open.*

eFigure1. Tip Sheet Used for Training

Tip Sheet

Start with a Generated Draft Reply

To give you a head start when responding to patient medical advice messages, In Basket now shows a Generated Draft Reply. Draft Replies are generated by a machine learning model that reviews the latest message sent by your patient and relevant patient details to predict how you might respond.

Draft Replies now appear for use in your Patient Medical Advice messages in In Basket. They are meant as a starting point for replies to your patients.

How are Draft Replies intended to be used?

- The drafted reply might not always provide appropriate advice and must not replace your clinical judgement. You must review all messages to verify that clinical advice and information is clinically appropriate for the patient and properly addresses their message.
- The model that generates the response is not intended to diagnose, prescribe treatment, or serve as a substitute for clinical judgment.
- It may not give full responses to patient messages which contain multiple questions or contain information that is not in patient's chart.
- Using the drafted text does not complete any other actions in the system. For example, you might need to reorder a medication for your patient.

Use a Generated Draft Reply to Respond to Messages

1. Open In Basket and select the Pt Advice Request folder. Open a message.
2. Review the message your patient has sent and the reply the model has drafted in the **Generated Draft Reply** section below the message.
3. Use the **Meds/Problems** and **Vitals/Labs** message report tabs to help you efficiently review the clinical accuracy of the draft reply.

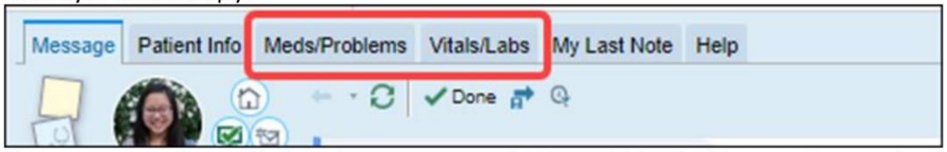

4. If you want to use the provided text in your response, click **Start with Draft**. This opens the normal reply window where you can edit the drafted reply to add more details or make it sound a bit more like you before sending the message.

© 2024 Tai-Seale M et al. *JAMA Network Open.*

5. If you don't like the provided text, you can click **Start Blank Reply,** use a QuickAction, or any other methods that you typically use to respond to the message.

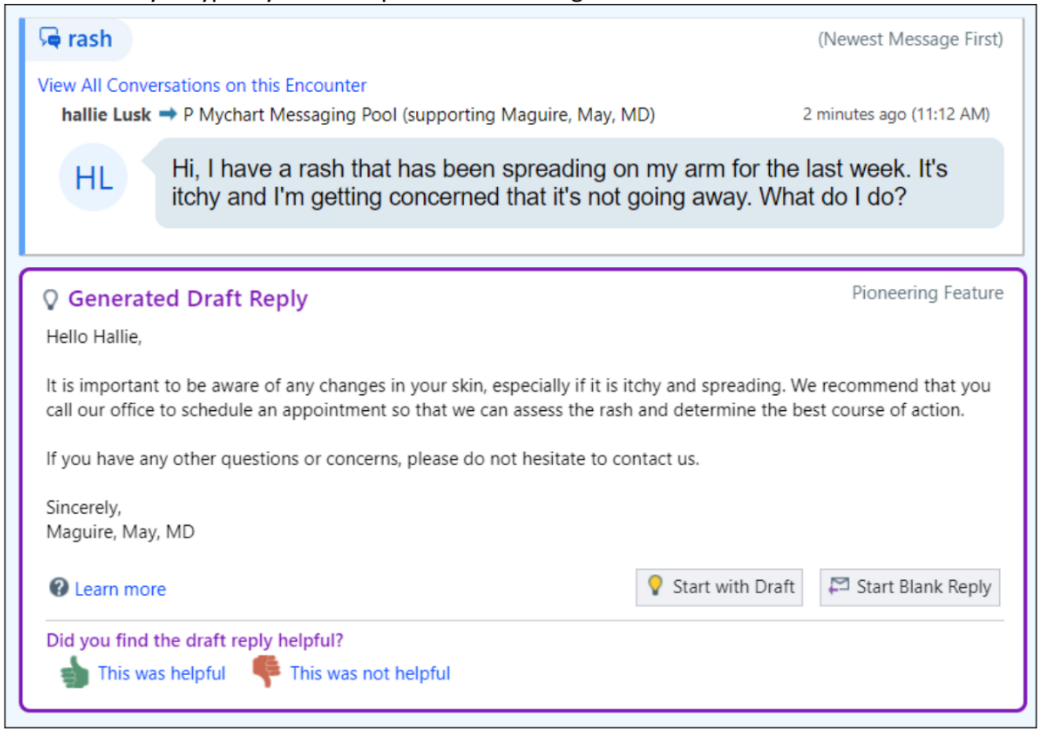

How is a Draft Reply Created?

In Basket uses a machine learning model to process a patient's message and generate text that might be appropriate as a response. In Basket sends the patient's message and relevant details as a prompt to the model and asks it to suggest a natural language response based on the information provided. Note that not all patient messages will have a generated draft reply. For example, given that not all messages make sense to reply to, the model might be unable to determine a useful response.

Learn More and Provide Feedback to Improve this Feature

- Click **Learn more** if you want to see a high-level overview of what a generated draft reply is and how they are created.
- Help Epic improve the model by providing feedback on whether a draft reply was helpful.
- If the draft was not helpful, let Epic know why.

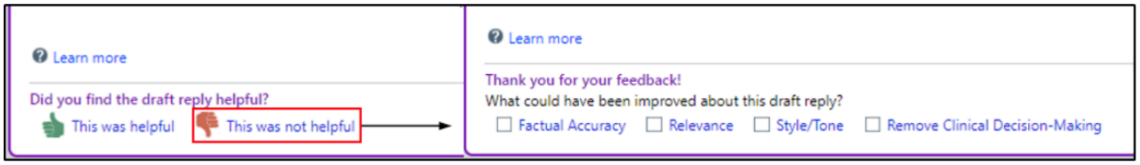

© 2024 Tai-Seale M et al. *JAMA Network Open.*

eFigure2. Screen Shots of a Generated Draft Reply and the Physician's Reply

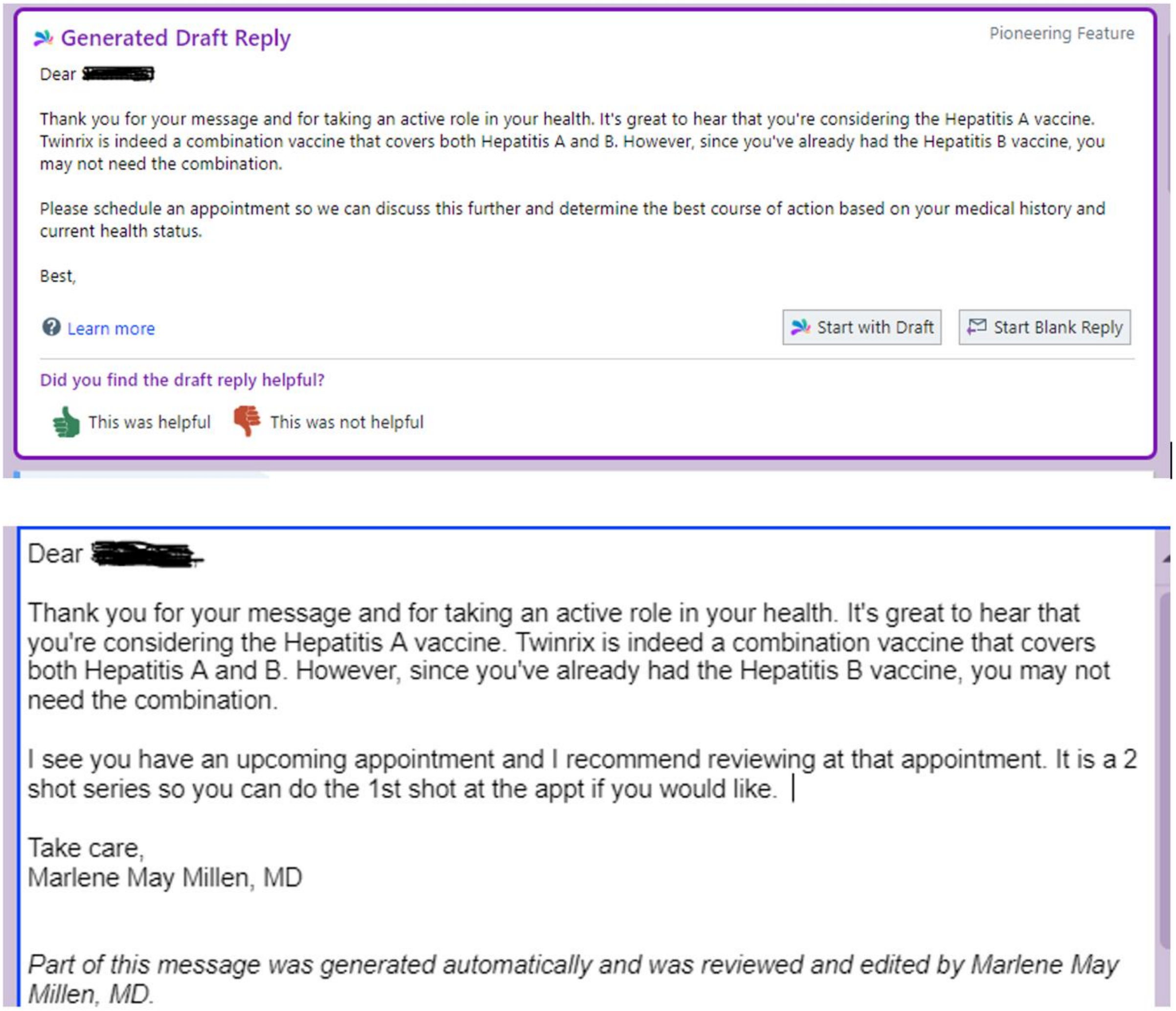

© 2024 Tai-Seale M et al. *JAMA Network Open.*

eMethods. Survey Questions in Post-Entry Survey

1. How likely are you to recommend using auto-generated draft replies to respond to patient messages to a colleague?

Not at all likely										Extremely likely
0	1	2	3	4	5	6	7	8	9	10
○	○	○	○	○	○	○	○	○	○	○

2. Explain the reason for your recommendation score.

© 2024 Tai-Seale M et al. *JAMA Network Open.*

eTable. Demographic Characteristics of Participants

	Immediate Arm	Delayed Arm	P-value[a]
Sex			0.56
- Female	18 (72.0)	17 (63.0)	
- Male	7 (28.0)	10 (37.0)	
Age (years)			0.52
- 25-34	8 (32.0)	4 (14.8)	
- 35-44	8 (32.0)	9 (33.3)	
- 45-54	4 (16.0)	9 (33.3)	
- 55-64	4 (16.0)	4 (14.8)	
- 65-74	1 (4.0)	1 (3.7)	
Employment Length (years)			0.37
- 1-5	9 (36.0)	6 (22.2)	
- 6-10	4 (16.0)	2 (7.4)	
- 11-15	3 (12.0)	9 (33.3)	
- 16-20	1 (4.0)	3 (11.1)	
- >20	6 (24.0)	6 (22.2)	
- N/A[b]	2 (8.0)	1 (3.7)	
Race/Ethnicity[c]			0.59
- Asian	7 (28.0)	7 (25.9)	
- Hispanic	0 (0.0)	1 (3.7)	
- White	16 (64.0)	14 (51.9)	
- Other	2 (8.0)	5 (18.5)	

Notes:

[a] P-values are from Fisher's exact test
[b] Declined to report
[c] "Other" includes "2 or more races" (n=4), "Prefer not to answer" (n=3)

© 2024 Tai-Seale M et al. *JAMA Network Open.*
