# Supplementary material for: AI-Generated Draft Replies Integrated Into Health Records and Physicians’ Electronic Communication
Source: JAMA Netw Open. 2024 Apr 15;7(4):e246565. doi: 10.1001/jamanetworkopen.2024.6565 (PMC11019394; doi:10.1001/jamanetworkopen.2024.6565)
Supplement: Supplement 2. — Data Sharing Statement [file jamanetwopen-e246565-s002.pdf]

Data Sharing Statement

Tai-Seale. AI-Generated Draft Replies Integrated Into Health Records and Physicians' Electronic Communication. *JAMA Netw Open*. Published April 15, 2024. doi:10.1001/jamanetworkopen.2024.6565

Data
Data available: No
